# Bidirectional Crosstalk between Stress-Induced Gastric Ulcer and Depression under Chronic Stress

**DOI:** 10.1371/journal.pone.0051148

**Published:** 2012-12-12

**Authors:** Shuang Zhang, Zhiwei Xu, Yan Gao, Yonghong Wu, Zhihui Li, Haifeng Liu, Chenggang Zhang

**Affiliations:** 1 Beijing Institute of Radiation Medicine, State Key Laboratory of Proteomics, Cognitive and Mental Health Research Center, Beijing, China; 2 Department of Gastroenterology, the General Hospital of Chinese People’s Armed Police Forces, Beijing, China; 3 School of Life Science, Anhui Medical University, Anhui, China; University of Tennessee, United States of America

## Abstract

Stress contributes to a variety of diseases and disorders such as depression and peptic ulcer. The present study aimed to investigate the correlation between stress ulcer and depression in pathogenesis and treatment by using chronic stress depression (CSD), chronic psychological stress ulcer (CPSU) and water immersion restrain stress models in rats. Our data showed that the ulcer index of the animals after CSD exposure was significantly higher than that of controls. Depression-like behaviors were observed in rat after CPSU exposure. Fluoxetine hydrochloride significantly reduced the ulcer index of rats exposed to CPSU stress, while ranitidine inhibited depression-like behavior of the animals in CSD group. The ulcer index of rats administered with mifepristone after CPSU stress was markedly reduced compared to CPSU group, although there was no significant difference in the depression-like behavior between mifepristone-treated CSD group and naive controls. We also found that the rats exposed to CPSU or CSD stress displayed a lower level of corticosterone than naive controls, however, the acute stress (AS) group showed an opposite result. Additionally, in order to study the relevance of H_2_ receptors and depression, we treated the CSD group with cimetidine and famotidine respectively. The data showed that cimetidine inhibited depression-like behavior in CSD rats, and famotidine had no impact on depression. Overall our data suggested that the hypothalamic-pituitary-adrenal (HPA) axis dysfunction may be the key role in triggering depression and stress ulcer. Acid-suppressing drugs and antidepressants could be used for treatment of depression and stress ulcer respectively. The occurrence of depression might be inhibited by blocking the central H_2_ receptors.

## Introduction

Hans Selye was a clinical endocrinologist and experimental biologist who focused his efforts on the study of stress responses [1]. He first introduced the concepts of stress, stressor, eustress, distress, and ‘General Adaptation Syndrome’ (GAS). He defined stress as the failed response of an individual to an emotional or physical threat [2]–[6]. Chronic physical and psychological stress has severe deleterious effects on an individual, and stress-related disorders have become a significant public health concern. The most common stress-related disorders impact the digestive system including stress ulcers and irritable bowel syndrome [7] as well as the central nervous system including psychiatric disorders such as depression and anxiety [8]. Although peptic ulcer was caused by disruption of the gastric mucosal defensive barrier [9], [10], depression is a complicated emotional and physical response of which the underlying mechanism was still not understood.

Factors inducing either stress ulcer or depression may contain surgery, trauma and emotional stress. The hypothalamic-pituitary-adrenal (HPA) axis is a major part of the neuroendocrine system that recognizes stresses and controls the reactions. The stress response of the HPA axis involves release of multiple hormones with diverse cognitive, physical, emotional and behavioral impacts. A survey of 104 patients with various cancers identified fluctuating corticosteroid level that was closely correlated with depression, pain and fatigue [11]. Gastric bleeding in rats exposed to acute water immersion and restraint is attributed to the decreased gastric pH and elevated adrenocorticotropic hormone (ACTH) levels [12], [13], suggesting the role of the HPA axis in stress ulcer formation and development. While multiple studies have been devoted to understand the neuroendocrine components of stress ulcers and depression, much remains unknown. Fundamental unanswered questions include whether the induction of stress ulcers and the onset of depression share a common etiology, and whether pharmacologic intervention of one stress-related disorder impacts the other or vice versa.

The H_2_ receptor antagonists are a class of drugs such as cimetidine (Cim), ranitidine, famotidine and nizatidine that inhibit gastric acid secretion from parietal cells, thereby protecting the gastric mucosa. However, the role of brain H_2_ receptors, specifically relating to depression and depression-like behaviors has not been disclosed yet. Histamine is known to activate HPA axis by affecting the H_2_ receptor in the brain, which leads to increase serum corticosterone level and induce the depression-like behaviors. Ranitidine, cimetidine and famotidine are H_2_ receptor antagonists widely used in clinical practice. In addition to their effects on the digestive system, ranitidine and cimetidine can pass across the blood brain barrier (BBB) and likely come into contact with the brain H_2_ receptors, but famotidine does not have the ability to gain access to the central nervous system by penetrating the BBB. In this scenario, the H_2_ receptor antagonists would serve to suppress depression-like behaviors. Supporting this hypothesis, clinical observations of the mental state of patients suffered from duodenal ulcer reported that the anxiety and depression scale was significantly improved compared to placebo after 4 weeks of treatment with ranitidine (*P*<0.05) [14]. Therefore, in the current study, we intended to illustrate the association of stress ulcer generation and the onset of depression utilizing the rat models of chronic stress. The results may provide novel strategies to the treatment for common chronic stress-induced disorders.

## Materials and Methods

### Animals

Adult male Sprague-Dawley (SD) rats weighing 200–220 g, were purchased from the Animal Center Laboratory (Laboratory Animal Centre, Academy of Military Medical Sciences). Every 9 animals were housed in polyethylene cages (485 mm × 350 mm × 200 mm) under standard housing conditions (12/12 h light-dark cycle starting at 7∶00 AM, temperature 22±2°C, free access to food and water) for 6–7 days before initiation of the experiment. All efforts were made to minimize animal suffering following the proposal of International Ethical Guideline for Biomedical Research (CIOMS/OMS, 1985).

### Treatments

Adult male SD rats were randomly divided into 10 groups (n = 9 per group): control group received saline (3 ml; p.o.); chronic psychological stress (ulcer forming; referred as CPSU; treated with saline, 3 ml; p.o.); chronic stress (leading to depression; CSD; treated with saline, 3 ml; p.o.); acute stress (AS); CPSU treated with ranitidine (histamine H_2_-receptor antagonist, 150 mg/kg, p.o.); CPSU treated with fluoxetine hydrochloride (selective serotonin re-uptake inhibitor, 1.8 mg/kg, p.o.); CPSU treated with mifepristone (glucocorticoid receptor antagonist, 65 mg/kg, p.o.); CSD treated with fluoxetine hydrochloride (1.8 mg/kg, p.o.); CSD treated with ranitidine (150 mg/kg, p.o.); and CSD treated with mifepristone (65 mg/kg, p.o.). A second set of studies was performed in which the SD rats were exposed to chronic stress depression in the presence of cimetidine (150 mg/kg, p.o. n = 9) and famotidine (150 mg/kg p.o. n = 9). Drugs were dissolved in saline. All drug solutions were freshly prepared and administrated 1 h prior to stress exposure.

### Chronic Psychological Stress Ulcer

Restraint was used to induce CPSU. Briefly, naive rats were placed in an adjustable acrylic hemi-cylindrical plastic tube (5 cm of diameter and 12 cm of length), as previously reported [15]. Rats were confined individually and exposed for a period of 4 h a day for consecutive 28 days. The major advantage of immobilization is that it produces both inescapable physical and psychological stress [16].

### Acute Stress

Rats were exposed to water immersion restraint stress (WIRS) where restrained in the stainless steel cages and immersed up to their xiphoid in a water bath maintained at 23±2°C. After the exposure for 4 h, the animals were sacrificed [17].

### Chronic Stress Depression

CSD was performed as described with some modifications [18]. Rats were exposed to one of nine randomly assigned stressors once daily for the next twenty-eight days. These involved food deprivation for 48 h; water deprivation for 24 h; tail pinch (1 cm apart from the end of the tail) for 5 min; overhang for 5 min; day and night reverse; 12°C cold water swimming for 5 min; shaking-crowding for 1 h; or 23±2°C water swimming for 10 min.

### Forced Swimming Test

Forced swimming test (FST) was performed as described [19]. Briefly, the experiment consisted of two forcing swim sessions. On the first day, the rats were individually placed in the testing cylinder (60 cm of the height and 30 cm of the diameter) filled to a 20 cm depth with water (25°C) for 15 min. The animals were placed in a plexiglas box for 30 min under a 60-W bulb to dry off following removal from the water and towel drying. Twenty-four hours after the first session, the animals were placed back in the cylinder individually for 6 min. The total period of immobility was recorded during the last 4 min of the test. The cylinders were emptied and cleaned between each animal.

### Sucrose Consumption Test

Animals were first treated to consume a palatable, weak (1%) sucrose solution. Training consisted of an initial 48 hours exposure to sucrose in place of water, followed by five 1-h tests in which sucrose was presented. On the day 28 following chronic stress, sucrose consumption was measured by determination of the mass of the bottle containing the sucrose solution before and after the test period. Sucrose preference was calculated as: (sucrose consumption)/(total fluid consumption) [20].

### Serum Corticosterone Analysis

Blood was collected from decapitated rats between 9∶00 AM and 11∶00 AM forty-eight hours after stress treatment and separated in a refrigerated centrifuge at 4°C (3,000 rpm × 10 min). Serum was stored at −80°C until the assays were performed. The serum corticosterone (CORT) was quantified by radio-immunoassay (RAI) according to the manufacturers protocols (PLA General Hospital, the RIA Technology Development Center, China).

### Ulcer Index Quantification

At the end of each experiment, animals were sacrificed by deep anesthesia with 10% chloral hydrate (350 mg/kg, i.p.). The stomachs were removed, inflated by injecting 2 ml of 1% formalin, and opened along the greater curvature. The area (mm^2^) of each hemorrhagic lesion was measured under a dissecting microscope with 10× magnification, summed per stomach and used as the lesion score.

### Statistics

Statistical analyses were performed using the SPSS 13.0 software. All data are presented as mean ± standard deviation. The Kolmogorov-Smirnov Z method was adopted for normal test, while the student’s *t*-test was used to identify significant differences between two samples. *P*<0.05 was recognized as statistically significant.

## Results

### Association of Stress Ulcers with Depression

**Figure 1:**
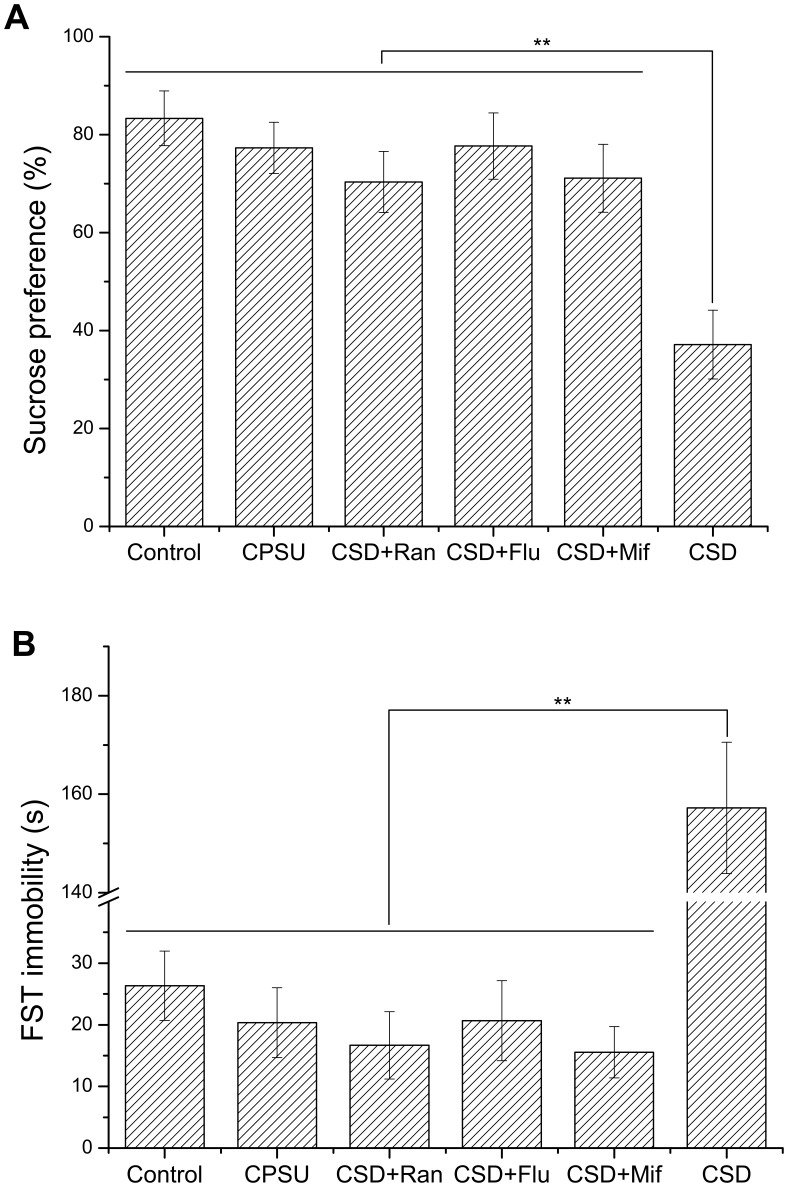
Effects of rat exposed to CPSU, CSD+Flu, CSD+Ran, CSD+Mif and CSD on sucrose preference of sucrose consumption test (SCT) (A) and immobility time of FST (B). The CPSU, CSD+Flu, CSD+Ran and CSD+Mif groups showed no significant difference on sucrose preference of the SCT test compared to control group. The sucrose preference of CSD animals was significantly decreased compared to control animals. The immobility time of CPSU, CSD+Flu, CSD+Ran and CSD+Mif groups showed no significant difference compared to the control group. The FST immobility time of CSD rats is significant higher than control animals. Flu: Fluoxetine hydrochloride; Ran: Ranitidine; Mif: Mifepristone. **P<0.01, n = 9 per group.

**Figure 2:**
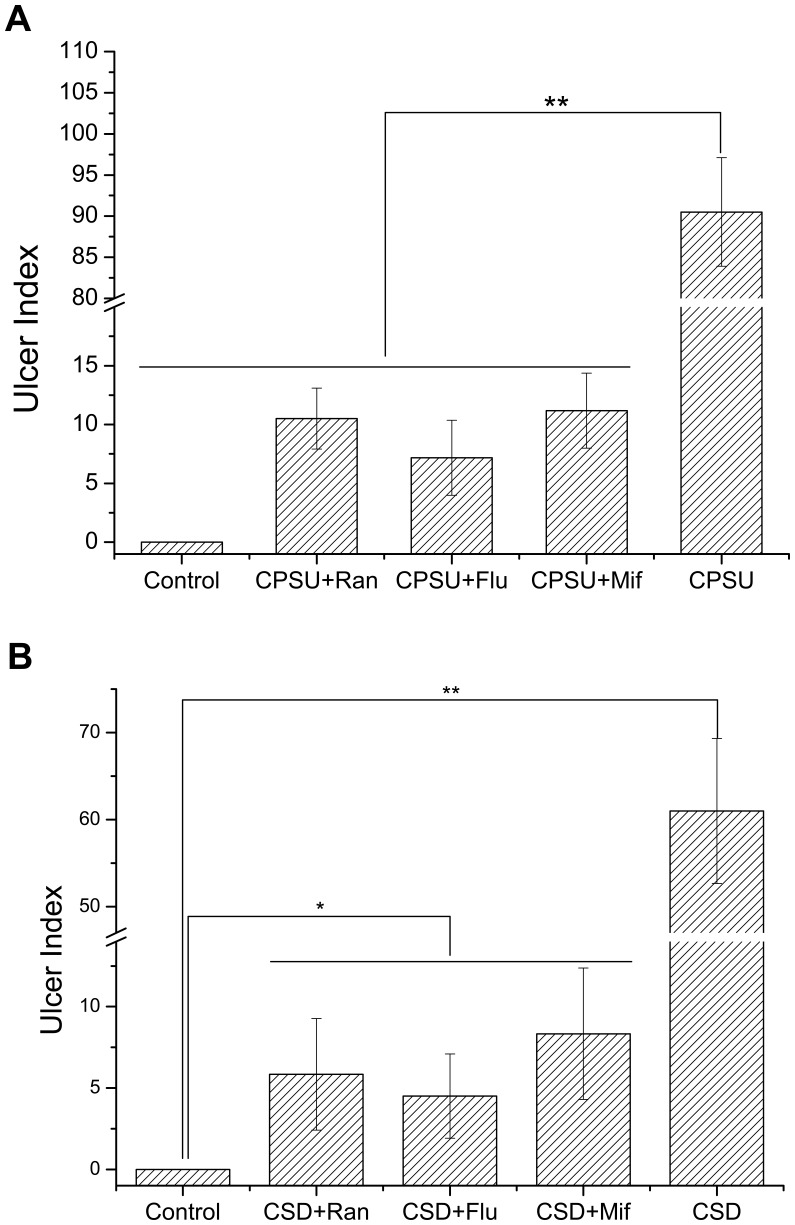
Effects of antagonists on ulcer index (UI) in CPSU procedure (A) and CSD procedure (B). The UIs in CPSU+Ran, CPSU+Flu and CPSU+Mif groups were significant lower than that of the CPSU treatment (A) and CSD treatment (B) groups. Flu: Fluoxetine hydrochloride; Ran: Ranitidine; Mif: Mifepristone. *P<0.05; ** *P*<0.01; n = 9 per group.

Initial studies were performed to determine whether a correlation exists between the generation of stress-associated ulcers and the onset of depression. The SD rats were exposed to a 28-day chronic psychological stress protocol. On day 28, rats were examined by the forced swimming test and sucrose consumption test (SCT). After completion of testing, the rats were sacrificed, serum was collected and ulcer index was quantified. The results of sucrose preference and immobility time of forced swimming test of the chronic psychological stress ulcer group showed no significant differences with the control group (*P*>0.05) (Fig. 1A). In contrast, scarring on the surface of the gastric mucosa was observed in rats following CPSU. Further analysis revealed diffuse congestion, erosion and bleeding of the glandular stomach, with an ulcer index significantly higher than that observed in the untreated control group (*P*<0.01) (Fig. 2A). In summary, the gastric mucosa of animals exposed to CPSU was significantly impaired, however no depression-like behavior was observed.

Rats exposed to CSD displayed significantly increased immobility time of forced swimming test compared to naive control animals as anticipated (*P*<0.01) (Fig. 1B). Similarly, sucrose preference was significantly lower in these rats compared to naive controls (*P*<0.01) (Fig. 1A). However, dotted and lineal erosion, bleeding and ulceration were clearly observed on the surface of the gastric mucosa in the rats exposed to CSD. Mucosal injury was observed primarily within the glandular stomach, and the ulcer index was significantly higher when compared with the control group (*P*<0.01) (Fig. 2B). In contrast to CPSU, CSD resulted in both depression-like behavior as well as gastric mucosal ulceration, suggesting that depression may be associated with ulcer formation in the animals.

### Antidepressants Inhibit Ulcer Formation in Both CPSU and CSD Models

The administration of the selective serotonin reuptake inhibitor (SSRI) antidepressant fluoxetine hydrochloride significantly reduced gastric mucosal impairment in rats with CPSU or CSD, compared to control animals (*P*<0.05) (Fig. 2). Fluoxetine hydrochloride completely eliminated gastric bleeding, with only a few scattered areas of hemorrhage and erosion were observed in the glandular stomach. The ulcer index of the fluoxetine-treated rats exposed to CPSU or CSD was significantly lower than rats that did not receive the medication (*P*<0.01 for each protocol) (Fig. 2A). These data indicate that administration of the antidepressant fluoxetine hydrochloride protects against stress-induced gastric mucosal injury.

### H_2_ Receptor Antagonists Impact Depression

Rats exposed to CSD were administered with ranitidine, a common H_2_ receptor antagonist, or placebo. Ranitidine inhibited depression-like behavior in CSD rats. No significant differences in the immobility time of forced swimming test or sucrose preference were observed from the CSD rats administered with ranitidine compared to naive control rats (*P*>0.05 for each test) (Fig. 1).

### Mifepristone Prevents Depression in Chronic Stress Models

In rats exposed to either CPSU or CSD, administration of the glucocorticoid receptor antagonist mifepristone reduced the ulcer index compared to CPSU treatment alone (*P*<0.01 for each) (Fig. 2). Interestingly, we did not observe significant differences in immobility time of the forced swimming test of mifepristone-treated rats exposed to CSD compared with naive controls (*P*>0.05) (Fig. 1B). In addition, we also did not observe any significant difference in the percentage of sucrose preference between mifepristone-treated and CSD exposed rats and the naive control group (*P*>0.05) (Fig. 1A). These results indicate that mifepristone may prevent the occurrence of depression-like behavior in rats.

### Serum Chemistry Following Stress

**Figure 3:**
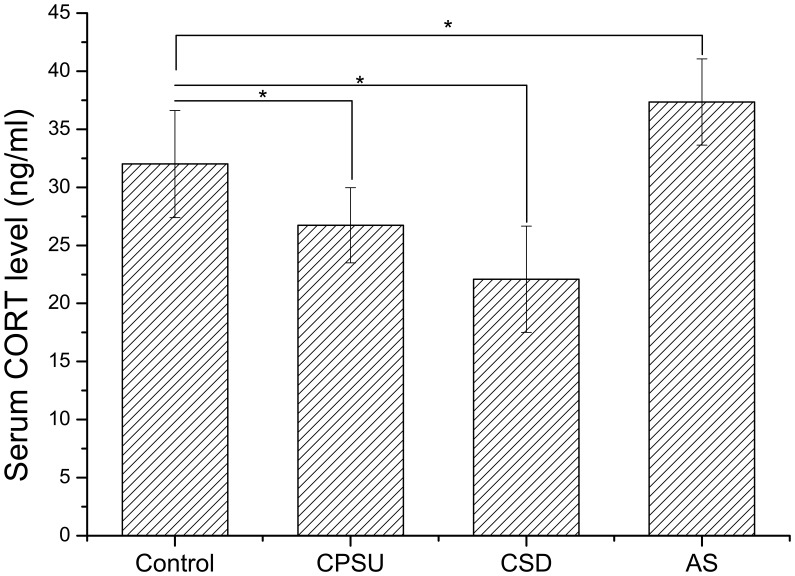
Serum corticosterone (CORT) level analysis of the animals received different treatments. The serum CORT levels of CPSU and CSD groups were markedly lower than control group. Acute stress (AS) resulted in a significant increase in the plasma CORT level compared to control group. * *P*<0.01, compared to control group. n = 9 per group.

Serum corticosterone level was measured following stress treatments. Rats exposed to acute stress displayed significantly higher corticosterone levels than naive controls (*P*<0.05) (Fig. 3). In contrast, rats exposed to either CPSU or CSD displayed significantly lower corticosterone than naive control rats (*P*<0.05) (Fig. 3).

### H_2_ Receptor Antagonists have Disparate Impacts on Depression-like Behaviors and Serum Corticosterone

**Figure 4:**
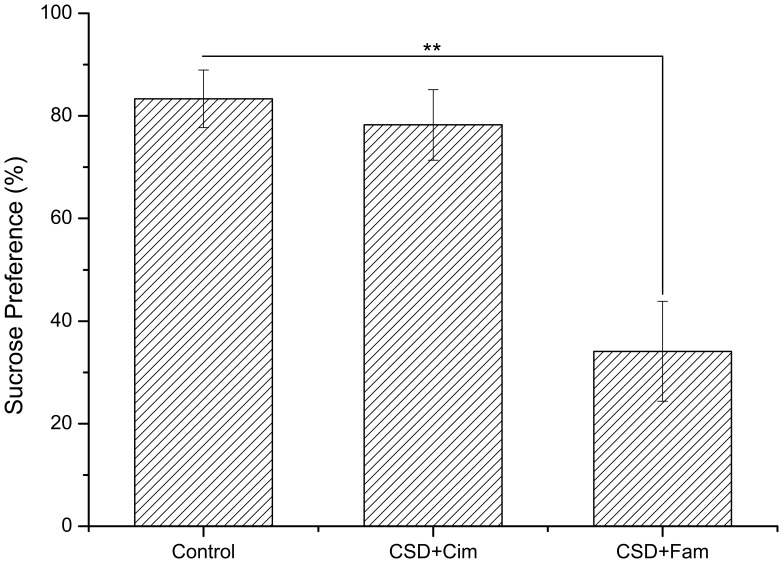
Effects of the histamine H_2_-receptor antagonist cimetidine and famotidine on sucrose preference of SCT test. CSD+Cim animals showed no significant difference compared to control group. The sucrose preference of CPSU+Fam animals was significant decreased than control animals. Cim: Cimetidine; Fam: Famotidine. ** *P*<0.01; n = 9 per group.

**Figure 5:**
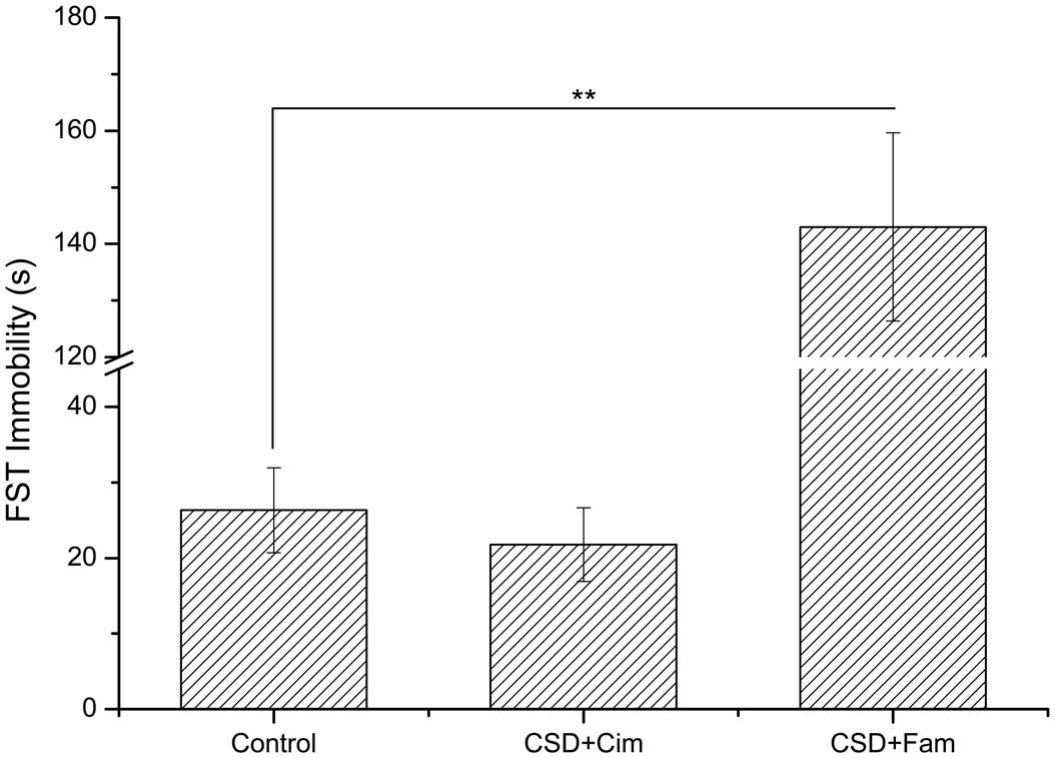
Effects of the histamine H_2_-receptor antagonist cimetidine and famotidine on immobility time of the FST test. The immobility time of CSD+Cim group showed no significant difference compared to the control animals. The FST immobility time of CSD+Fam animals was significant higher than control animals. Cim: Cimetidine; Fam: Famotidine. ** *P*<0.01; n = 9 per group.

**Figure 6:**
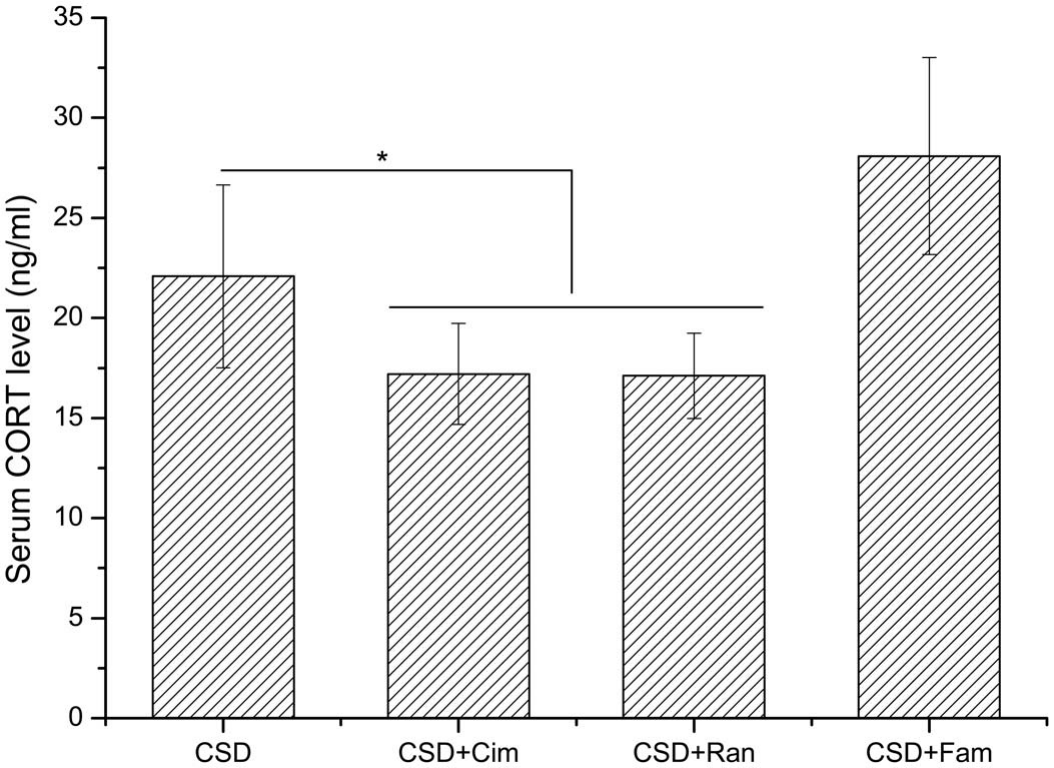
Effects of the histamine H_2_-receptor antagonists cimetidine and famotidine on serum CORT level. The serum CORT level of CSD+Fam exposure showed no significant difference compared to the CSD group. In CSD+Ran and CSD+Cim animals, the serum CORT level was lower than control rats. Cim: Cimetidine; Ran: Ranitidine; Fam: Famotidine. * *P*<0.05; n = 9 per group.

No significant differences in the immobility time of forced swimming test (*P*>0.05) or the percentage of sugar preference were observed between the cimetidine-treated rats with CSD and naive controls as shown in Figure 4 and Figure 5 (*P*>0.05). However, the immobility time of forced swimming test of the famotidine-treated rats was significantly increased (*P*<0.01), while the percentage of sucrose preference in this group was significantly decreased compared to naive control rats as shown in Figure 4 and Figure 5 (*P*<0.01). In summary, these data suggest that the H_2_ receptor antagonist, cimetidine, which is able to pass across the BBB, has a significant inhibitive effect on depression-like behavior; while famotidine, which cannot cross the BBB, does not offer impact on depression. Serum chemistry analysis revealed that no significant differences in corticosterone levels between famotidine-treated rats and rats with CSD as shown in Figure 6 (*P*>0.05). However, serum corticosterone levels in CSD-exposed rats treated with ranitidine or cimetidine were significantly lower than these in control group alone with CSD (*P*<0.05) (Fig. 6).

**Figure 7:**
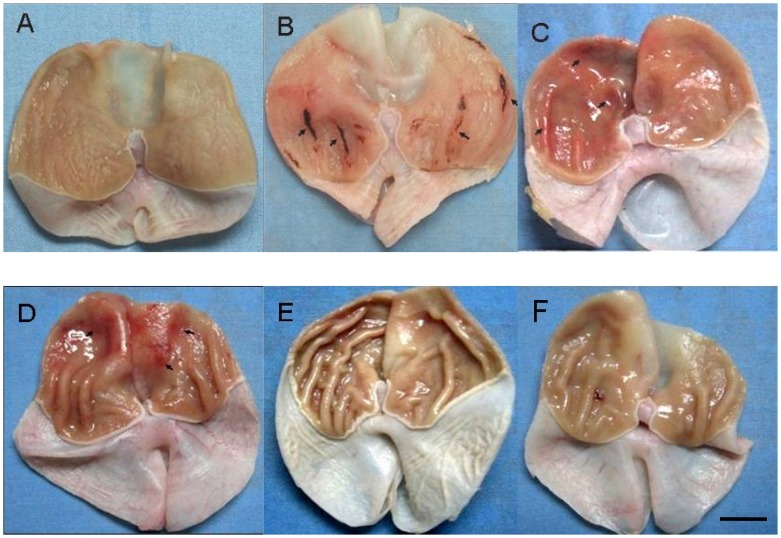
Diffuse congestion, erosion and bleeding at the glandular stomach mucosa of the SD rats. A: Control; B: Acute stress; C: Chronic psychological stress ulcer; D: Chronic stress depression; E: Chronic stress depression treated with fluoxetine hydrochloride; F: Chronic stress depression treated with ranitidine. Scale bar = 3 mm.

In order to have a better understanding on the morphological changes of different groups with treatments, the diffuse congestion, erosion and bleeding at the glandular stomach mucosa of the SD rats could be seen in Figure 7.

## Discussion

Stress-related disorders have become more important and severe in current society. There is increasing interest in deciphering the role of stress-induced factors in stress ulcer and depression. In this study, we found the bidirectional crosstalk between stress ulcer disease and major depression as well as the common mechanism of pathogenesis.

It is known that the H_2_ receptor antagonists are a class of drugs inhibiting gastric acid secretion commonly used in clinical practice, which blocking the H_2_ receptors on parietal cells plays a role in protecting the gastric mucosa. Ranitidine, cimetidine and famotidine are widely used H_2_ receptor antagonists. It has been demonstrated that no depression-like behaviors are presented in ranitidine-treated rats with CSD; therefore we speculate that H_2_ receptors in the brain plays an important role in the process of the occurrence and development of depression, consistent with previous reports [21]. Supplementary interventions with cimetidine and famotidine were conducted for further validation. The data showed that cimetidine which can easily go across the blood-brain barrier had a significant suppressing effect on depression-like behaviors but not for famotidine. As famotidine has a poor lipophilicity, its ability to penetrate the blood-brain barrier is weaker than the other H_2_ receptor antagonists. It is suggested that the efficacy on depression- behaviors of cimetidine and ranitidine is related to the H_2_ receptor in the brain. Although the mechanism still remains unclear, it has been reported that H_2_ receptor antagonist does prevent the occurrence of depression. To understand the underlying mechanism of this phenomenon, we examined the serum corticosterone level of rats exposed to CSD and treated with ranitidine or cimetidine which is significantly lower than that of the rats exposed to CSD and receiving placebo. Cerebral histamine is produced by mastocytes which can activate the HPA axis after stimulation [22]. It is possible that the H_2_ receptor antagonists such as ranitidine and cimetidine may affect histamine to activate HPA axis through blocking the H_2_ receptor in the brain, which decrease serum corticosterone level and suppress the depression-like behaviors. Chandishwar Nath found that central histamine plays a facilitator role in occurrence of depression in animal. Administration with histamine can enhance immobility in the swimming despair test in mice [21]. Some researchers concluded that central histamine H_2_ receptor facilitates aggressive behavior [23]. It seemed that the H_2_ receptor antagonists may play an important role in suppressing the depression-like behaviors via reducing HPA axis activation and inhibiting histamine by blocking the H_2_ receptor in the central nervous system. Thus our data provides new clues to the mechanism of depression for further in-depth study.

A pilot study has been conducted which showed that erosion and bleeding in CSD group was observed in glandular stomach mucosa with ulcer index significantly higher than the control group, and depression behavior test revealed a significant depression-like behavior. Our results indicated that the rats with depression-like behavior suffered from a severe gastric mucosal impairment. Meanwhile depression-like behavior in the CPSU group was not statistically different from the control group, suggesting that the stress ulcer is not necessarily accompanied by the occurrence of depression. The results are consistent with previous studies [24], [25], although other authors reported a different result [26]. It is known that depression is related to defective function of 5-hydroxytryptamine. Chronic restraint stress is associated with enhanced postsynaptic behavioral sensitivity to the 5-hydroxytryptamine agonist 5-methoxy-N, N-dimethyltryptamine [24], [25]. Susceptibility to chronic stress is affected by multiple factors such as circadian rhythms, age, gender, strain or genomic makeup [27]–[30]. In conclusion, it is believed that certain relevance does exist between stress ulcer and depression, and that depression often goes with stress ulcer while stress ulcer is not necessarily accompanied by the occurrence of depression.

**Figure 8:**
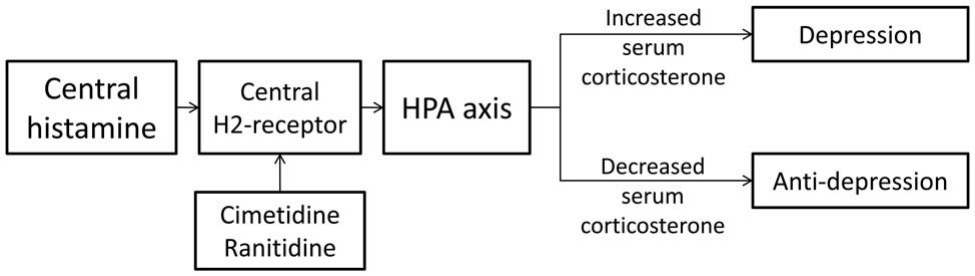
Diagram showing the depressant action of H_2_ receptor antagonists.

Previous study indicated that repeated pretreatment with antidepressant citalopram exhibited a significant gastroprotective efficacy in cold restraint model [31]. Antidepressants have been thought to relieve gastric mucosa impairment via reducing gastric acid secretion and repairing the mucus-bicarbonate barrier. Suleyman identified that administration of antidepressants increased expression of antioxidant indicators including glutathione and superoxide dismutase, while decreasing the expression of oxidative indicators such as hydrogen peroxide, malondialdehyde and myeloperoxidase [32]. Fluoxetine is believed to act as a SSRI and frequently used to treat depression [33]. It could increase cortical gamma-aminobutyric acid (GABA) levels in stressed rats [34], and gastroprotective activity of GABA appears to be mediated by increasing the gastric mucosal blood flow that was depended on sensory neuron and NO systems [35]. A more plausible mechanism was presented by Kanof, suggesting that antidepressants could protect gastric mucosa via interactions with H_2_ receptors in the brain [36]. As the H_2_ receptor antagonists that pass across the BBB were able to impact depression, our data also provided further evidence for this issue. Embracing all these possibilities, a possible mechanism was proposed in Figure 8.

Stressors activate the paraventricular nucleus, giving rise to the release of corticotropin releasing hormone (CRH). CRH acts on the pituitary and stimulates the secretion of ACTH which promotes glucocorticoids release from the adrenal cortex [37]. Glucocorticoids are the end-product of the HPA axis referred to as cortisol in human and corticosterone in rodents [38]. The causal relationship between glucocorticoids and depression remains unclear. Researches on whether stressors will induce the release of glucocorticoids are inconsistent. Some studies suggest that stress triggers depression with increasing output of glucocorticoids [39], [40]. In contrast to these papers, another theory considers that stress-induced decline in glucocorticoids output involves in the pathogenesis of depression [41]. Our results demonstrated the level of corticosterone was significantly increased in rats exposed to AS, on the contrary the rats exposed to chronic stress (CPSU or CSD) showed obviously lower level of corticosterone than naive controls. This situation could be considered as the influence of timing on the HPA axis. Sanne H Booij pointed out that “when chronic stress first begins, there is an initial activation of the HPA axis, which results in elevated concentrations of ACTH and cortisol. However, the findings suggest that as time passes, this activity diminishes, and cortisol secretion rebounds to below normal” [42]. Glucocorticoids are released in response to many stressors as a protective hormone, the effect of which is the normalization of stress response, serving as adaptive factors to stress events or circumstance. Investigations showed that the experience of repeated or chronic stress can result in hypo-active state of the HPA axis [43]. We speculated that the decline in serum corticosterone level may represent an adaptive response to stress. In addition, high level of glucocorticoids can be induced by stress, which increases the sensitivity to the negative feedback in pituitary [44], [45]. There is general agreement that large quantities of glucocorticoids may induce or aggravate peptic ulcer. Glucocorticoids can not only interfere with tissue repair, elevate levels of gastric acid and pepsin, but also reduce the secretion of gastric mucus, and eventually impair gastric mucosal barrier resulting in peptic ulcer. Moreover depression is characterized with reduced neurogenesis, mainly in hippocampus [46]. The elevation of glucocorticoids can interfere by a variety of adverse structural and cellular changes including neuronal damage in hippocampus [47], cause increased secretion of hypothalamic CRH, and result in impaired reactivity to stress [42], [48]. Clearly neuroendocrine system that regulates stress response is extremely complex. Our data suggest the HPA axis is significantly involved in chronic stress-induced ulcer formation and the onset of depression via multiple mechanisms, among which it mediates the structural and cellular changes in hippocampus, and consequently the alterations induce hippocampus damage, which may increase the risk of depression. Furthermore, these data indicate that intervention at the level of the HPA axis may provide broad protection from chronic stress disorder.

The above experiments have confirmed that stress ulcer does correlate with depression: antidepressants can probably be used for the treatment of stress ulcer and acid-suppressing drugs can be used to treat depression, suggesting that these two diseases may share a common mechanism of pathogenesis. Mifepristone is a glucocorticosteroid receptor and progesterone receptor antagonist currently being tested as potential antidepressant [49]. We used mifepristone for better understanding the interaction between stress ulcer and depression on the HPA axis. Our data demonstrated that rats administered with mifepristone in the two groups (CPSU and CSD) did not suffer from severe gastric mucosal damage. Compared with the control group, there was no difference in depression-like behavior in the CSD group, which was given mifepristone. These findings suggested that mifepristone significantly decreases depression-like behavior and the occurrence of stress ulcer in chronic stressed rats, consistent with an antidepressant effect. Previous study suggested that mifepristone ameliorate acute stress-induced depression-like behavior by targeting the glucocorticoid receptor [50]. Since the chronic stressors in the CPSU and CSD animal models could be understood as the integration of different acute stressors, mifepristone blocks the glucocorticoid receptor and invalidates the end-product of the HPA axis, and eventually exerts a positive effect on reducing depression-like behavior and protecting the gastric mucosa. The results have shown that glucocorticoid plays an important role in the occurrence of stress ulcer and depression by which we can conclude that interaction of these two diseases does exist on the HPA axis.

### Conclusions

There is a certain relationship between stress ulcer and depression. Stress ulcer is not necessarily accompanied by the occurrence of depression while depression often goes with stress ulcer. Antidepressants and acid-suppressing drugs may probably be used for the treatment of stress ulcer and depression respectively. Acute stress actives the HPA axis and chronic stress may result in reduced HPA axis activation. Interaction exists between the occurrence of depression and stress ulcer on the HPA axis, meanwhile the H_2_-receptor in the brain plays an important role in depression.

## References

[1] Perdrizet GA (1997) Hans Selye and beyond: responses to stress. Cell Stress Chaperones 2: 214–219. 9495278 10.1379/1466-1268(1997)002<0214:hsabrt>2.3.co;2 PMC313000

[2] Selye H (1998) A syndrome produced by diverse nocuous agents. 1936. J Neuropsychiatry Clin Neurosci 10: 230–231. 9722327 10.1176/jnp.10.2.230a

[3] Selye H (1946) The general adaptation syndrome and the diseases of adaptation. J Allergy 17: 231 passim. 10.1016/0021-8707(46)90148-7 20990814

[4] Selye H (1954) Sketch for a unified theory of medicine. Int Rec Med Gen Pract Clin 167: 181–203. 13162615

[5] Selye H (1965) The Stress of Life–New Focal Point for Understanding Accidents. Nurs Forum 4: 28–38. 14280643 10.1111/j.1744-6198.1965.tb01011.x

[6] Selye H (1964) The Stress of Life–New Focal Point for Understanding Accidents. Ind Med Surg 33: 621–625. 14216330

[7] Murison R (2001) Is there a role for psychology in ulcer disease? Integr Physiol Behav Sci 36: 75–83. 11484998 10.1007/BF02733948

[8] McKernan DP, Dinan TG, Cryan JF (2009) “Killing the Blues”: a role for cellular suicide (apoptosis) in depression and the antidepressant response? Prog Neurobiol 88: 246–263. 19427352 10.1016/j.pneurobio.2009.04.006

[9] Kato A, Naitou H, Namioka M, Akimoto M, Ishii T, et al (2010) Proteomic identification of serum proteins associated with stress-induced gastric ulcers in fasted rats. Biosci Biotechnol Biochem 74: 812–818. 20378983 10.1271/bbb.90897

[10] Brzozowska I, Ptak-Belowska A, Pawlik M, Pajdo R, Drozdowicz D, et al (2009) Mucosal strengthening activity of central and peripheral melatonin in the mechanism of gastric defense. J Physiol Pharmacol 60 Suppl 747–56. 20388945

[11] Thornton LM, Andersen BL, Blakely WP (2010) The pain, depression, and fatigue symptom cluster in advanced breast cancer: covariation with the hypothalamic-pituitary-adrenal axis and the sympathetic nervous system. Health Psychol 29: 333–337. 20496988 10.1037/a0018836 PMC2910549

[12] Verma P, Hellemans KG, Choi FY, Yu W, Weinberg J (2010) Circadian phase and sex effects on depressive/anxiety-like behaviors and HPA axis responses to acute stress. Physiol Behav 99: 276–285. 19932127 10.1016/j.physbeh.2009.11.002 PMC2856664

[13] Fraites MJ, Cooper RL, Buckalew A, Jayaraman S, Mills L, et al (2009) Characterization of the hypothalamic-pituitary-adrenal axis response to atrazine and metabolites in the female rat. Toxicol Sci 112: 88–99. 19710361 10.1093/toxsci/kfp194

[14] Robins AH, Lucke W, McFadyen ML, Wright JP (1984) Effect of the H2-receptor antagonist ranitidine on depression and anxiety in duodenal ulcer patients. Postgrad Med J 60: 353–355. 6330716 10.1136/pgmj.60.703.353 PMC2417877

[15] Rai D, Bhatia G, Sen T, Palit G (2003) Anti-stress effects of Ginkgo biloba and Panax ginseng: a comparative study. J Pharmacol Sci 93: 458–464. 14737017 10.1254/jphs.93.458

[16] Madrigal JL, Olivenza R, Moro MA, Lizasoain I, Lorenzo P, et al (2001) Glutathione depletion, lipid peroxidation and mitochondrial dysfunction are induced by chronic stress in rat brain. Neuropsychopharmacology 24: 420–429. 11182537 10.1016/S0893-133X(00)00208-6

[17] Brzozowski T, Zwirska-Korczala K, Konturek PC, Konturek SJ, Sliwowski Z, et al (2007) Role of circadian rhythm and endogenous melatonin in pathogenesis of acute gastric bleeding erosions induced by stress. J Physiol Pharmacol 58 Suppl 653–64. 18212400

[18] Katz RJ, Roth KA, Carroll BJ (1981) Acute and chronic stress effects on open field activity in the rat: implications for a model of depression. Neurosci Biobehav Rev 5: 247–251. 7196554 10.1016/0149-7634(81)90005-1

[19] Porsolt RD, Anton G, Blavet N, Jalfre M (1978) Behavioural despair in rats: a new model sensitive to antidepressant treatments. Eur J Pharmacol 47: 379–391. 204499 10.1016/0014-2999(78)90118-8

[20] D’Aquila PS, Brain P, Willner P (1994) Effects of chronic mild stress on performance in behavioural tests relevant to anxiety and depression. Physiol Behav 56: 861–867. 7824585 10.1016/0031-9384(94)90316-6

[21] Nath C, Gulati A, Dhawan KN, Gupta GP (1988) Role of central histaminergic mechanism in behavioural depression (swimming despair) in mice. Life Sci 42: 2413–2417. 2967413 10.1016/0024-3205(88)90339-6

[22] Gadek-Michalska A, Chlap Z, Turon M, Bugajski J, Fogel WA (1991) The intracerebroventricularly administered mast cells degranulator compound 48/80 increases the pituitary-adrenocortical activity in rats. Agents Actions 32: 203–208. 1862742 10.1007/BF01980874

[23] Nath C, Gulati A, Dhawan KN, Gupta GP, Bhargava KP (1982) Evidence for central histaminergic mechanism in foot shock aggression. Psychopharmacology (Berl) 76: 228–231. 6808541 10.1007/BF00432550

[24] Kennett GA, Dickinson SL, Curzon G (1985) Enhancement of some 5-HT-dependent behavioural responses following repeated immobilization in rats. Brain Res 330: 253–263. 4039215 10.1016/0006-8993(85)90684-5

[25] Kennett GA, Dickinson SL, Curzon G (1985) Central serotonergic responses and behavioural adaptation to repeated immobilisation: the effect of the corticosterone synthesis inhibitor metyrapone. Eur J Pharmacol 119: 143–152. 4092729 10.1016/0014-2999(85)90290-0

[26] Mei X, Xu D, Xu S, Zheng Y (2011) Gastroprotective and antidepressant effects of a new zinc(II)-curcumin complex in rodent models of gastric ulcer and depression induced by stresses. Pharmacol Biochem Behav 99: 66–74. 21513730 10.1016/j.pbb.2011.04.002

[27] Gorka Z, Moryl E, Papp M (1996) Effect of chronic mild stress on circadian rhythms in the locomotor activity in rats. Pharmacol Biochem Behav 54: 229–234. 8728562 10.1016/0091-3057(95)02173-6

[28] Pardon M, Perez-Diaz F, Joubert C, Cohen-Salmon C (2000) Age-dependent effects of a chronic ultramild stress procedure on open-field behaviour in B6D2F1 female mice. Physiol Behav 70: 7–13. 10978471 10.1016/s0031-9384(00)00216-x

[29] Bielajew C, Konkle AT, Kentner AC, Baker SL, Stewart A, et al (2003) Strain and gender specific effects in the forced swim test: effects of previous stress exposure. Stress 6: 269–280. 14660059 10.1080/10253890310001602829

[30] Pothion S, Bizot JC, Trovero F, Belzung C (2004) Strain differences in sucrose preference and in the consequences of unpredictable chronic mild stress. Behav Brain Res 155: 135–146. 15325787 10.1016/j.bbr.2004.04.008

[31] Saxena B, Singh S (2011) Investigations on gastroprotective effect of citalopram, an antidepressant drug against stress and pyloric ligation induced ulcers. Pharmacol Rep 63: 1413–1426. 22358089 10.1016/s1734-1140(11)70705-8

[32] Suleyman H, Cadirci E, Albayrak A, Polat B, Halici Z, et al (2009) Comparative study on the gastroprotective potential of some antidepressants in indomethacin-induced ulcer in rats. Chem Biol Interact 180: 318–324. 19497431 10.1016/j.cbi.2009.03.002

[33] Mennigen JA, Lado WE, Zamora JM, Duarte-Guterman P, Langlois VS, et al (2010) Waterborne fluoxetine disrupts the reproductive axis in sexually mature male goldfish, Carassius auratus. Aquat Toxicol 100: 354–364. 20864192 10.1016/j.aquatox.2010.08.016

[34] Abdel-Sater KA, Abdel-Daiem WM, Sayyed Bakheet M (2012) The gender difference of selective serotonin reuptake inhibitor, fluoxetine in adult rats with stress-induced gastric ulcer. Eur J Pharmacol 688: 42–48. 22546225 10.1016/j.ejphar.2012.04.019

[35] Harty RF, Ancha HR, Xia Y, Anderson M, Jazzar A (2004) GABAergic mechanisms of gastroprotection in the rat: role of sensory neurons, prostaglandins, and nitric oxide. Dig Dis Sci 49: 1875–1881. 15628719 10.1007/s10620-004-9586-z

[36] Kanof PD, Greengard P (1978) Brain histamine receptors as targets for antidepressant drugs. Nature 272: 329–333. 634355 10.1038/272329a0

[37] Herman JP, Cullinan WE (1997) Neurocircuitry of stress: central control of the hypothalamo-pituitary-adrenocortical axis. Trends Neurosci 20: 78–84. 9023876 10.1016/s0166-2236(96)10069-2

[38] Suri D, Vaidya VA (2012) Glucocorticoid regulation of BDNF: Relevance to hippocampal structural and functional plasticity. Neuroscience. 10.1016/j.neuroscience.2012.08.065 22967840

[39] Gourley SL, Kiraly DD, Howell JL, Olausson P, Taylor JR (2008) Acute hippocampal brain-derived neurotrophic factor restores motivational and forced swim performance after corticosterone. Biol Psychiatry 64: 884–890. 18675955 10.1016/j.biopsych.2008.06.016 PMC2633780

[40] Gourley SL, Wu FJ, Kiraly DD, Ploski JE, Kedves AT, et al (2008) Regionally specific regulation of ERK MAP kinase in a model of antidepressant-sensitive chronic depression. Biol Psychiatry 63: 353–359. 17889834 10.1016/j.biopsych.2007.07.016 PMC2277331

[41] Rybakina EG, Shanin SN, Fomicheva EE, Korneva EA (2009) Cellular and molecular mechanisms of interaction between the neuroendocrine and immune systems under chronic fatigue syndrome in experiment. Ross Fiziol Zh Im I M Sechenova 95: 1324–1335. 20141043

[42] Booij SH, Bouma EM, de Jonge P, Ormel J, Oldehinkel AJ (2012) Chronicity of depressive problems and the cortisol response to psychosocial stress in adolescents: The TRAILS study. Psychoneuroendocrinology. 10.1016/j.psyneuen.2012.08.004 22963816

[43] Miller GE, Chen E, Zhou ES (2007) If it goes up, must it come down? Chronic stress and the hypothalamic-pituitary-adrenocortical axis in humans. Psychol Bull 133: 25–45. 17201569 10.1037/0033-2909.133.1.25

[44] Fries E, Hesse J, Hellhammer J, Hellhammer DH (2005) A new view on hypocortisolism. Psychoneuroendocrinology 30: 1010–1016. 15950390 10.1016/j.psyneuen.2005.04.006

[45] Dickens M, Romero LM, Cyr NE, Dunn IC, Meddle SL (2009) Chronic stress alters glucocorticoid receptor and mineralocorticoid receptor mRNA expression in the European starling (Sturnus vulgaris) brain. J Neuroendocrinol 21: 832–840. 19686439 10.1111/j.1365-2826.2009.01908.x

[46] Jacobs BL, van Praag H, Gage FH (2000) Adult brain neurogenesis and psychiatry: a novel theory of depression. Mol Psychiatry 5: 262–269. 10889528 10.1038/sj.mp.4000712

[47] Sapolsky RM, Uno H, Rebert CS, Finch CE (1990) Hippocampal damage associated with prolonged glucocorticoid exposure in primates. J Neurosci 10: 2897–2902. 2398367 10.1523/JNEUROSCI.10-09-02897.1990 PMC6570248

[48] Jacobson L, Sapolsky R (1991) The role of the hippocampus in feedback regulation of the hypothalamic-pituitary-adrenocortical axis. Endocr Rev 12: 118–134. 2070776 10.1210/edrv-12-2-118

[49] Belanoff JK, Flores BH, Kalezhan M, Sund B, Schatzberg AF (2001) Rapid reversal of psychotic depression using mifepristone. J Clin Psychopharmacol 21: 516–521. 11593077 10.1097/00004714-200110000-00009

[50] Wulsin AC, Herman JP, Solomon MB (2010) Mifepristone decreases depression-like behavior and modulates neuroendocrine and central hypothalamic-pituitary-adrenocortical axis responsiveness to stress. Psychoneuroendocrinology 35: 1100–1112. 20149549 10.1016/j.psyneuen.2010.01.011 PMC3934351

